# Thalamic Bursts Down-regulate Cortical Theta and Nociceptive Behavior

**DOI:** 10.1038/s41598-017-02753-6

**Published:** 2017-05-30

**Authors:** Brian W. LeBlanc, Brent Cross, Kelsey A. Smith, Catherine Roach, Jimmy Xia, Yu-Chieh Chao, Joshua Levitt, Suguru Koyama, Christopher I. Moore, Carl Y. Saab

**Affiliations:** 1 Department of Neurosurgery, Rhode Island Hospital, Providence, RI USA; 2 Department of Neuroscience, Brown University, Providence, RI USA; 3 Department of Anesthesiology, Beijing Chaoyang Hospital, Capital Medical University, Beijing, China; 4 Center for Biomedical Engineering, Brown University, Providence, RI USA; 5 Laboratory for Pharmacology, Asahi KASEI Pharma Corporation, Shizuoka, Japan

## Abstract

We tested the relation between pain behavior, theta (4–8 Hz) oscillations in somatosensory cortex and burst firing in thalamic neurons *in vivo*. Optically-induced thalamic bursts attenuated cortical theta and mechanical allodynia. It is proposed that thalamic bursts are an adaptive response to pain that de-synchronizes cortical theta and decreases sensory salience.

## Introduction

Evidence for augmented theta (4–8 Hz) oscillations in primary somatosensory (SI) cortex was initially reported in chronic pain patients using electroencephalography (EEG)^1^, followed by data in animal pain models using EEG^2^, electrocorticography^3^ and intracortical local field potential (LFP) recordings^4^. Excess theta power is attenuated upon pain relief following thalamic lesion in humans^5^ and analgesic drug treatments in animal models^2, 4^. Though generators of theta oscillations remain unidentified, sensory thalamus is a likely contributor^4, 6^ given its unique ability to gate sensory information and to modulate cortical oscillations required for optimal behavioral responses^7^.

Thalamocortical neurons fire in two dynamic and state dependent modes: tonic and high frequency or ‘burst’ discharges of two or more action potentials^8^. The respective roles of these modes in gating sensory processing remains controversial^9, 10^. Burst patterns have been characterized pre-clinically^11–13^ and clinically^14, 15^ during pain states. However, conflicting evidence suggests thalamic bursts may be positively^4, 6, 11–14^ or negatively correlated with pain^16–20^. Thalamic bursts are triggered predominantly by GABAergic drive from reticular thalamic nucleus (TRN), a thin layer overlaying thalamus that receives strong input from limbic cortical areas conveying information related to emotion and attention^21^. Pharmacologic and molecular data further suggest that the GABA-mediated inhibitory tone in thalamus is suppressed during pain^22, 23^, presumably due to inhibition of TRN neurons^24, 25^.

We tested the hypothesis that rescuing TRN’s ‘gating’ function by selective optical stimulation releases TRN neurons from inhibition, thus promoting thalamic bursting, reducing cortical theta and reversing nociceptive behavior. We reasoned that a multidisciplinary approach combining electrophysiology, optogenetics and behavior to probe this important but under-appreciated question in a longitudinal study design would provide the strongest evidence to date for a *causal* spatiotemporal relation between micro-scale unitary bursting in thalamus and macro-scale LFP oscillations in cortex.

**Figure 1:**
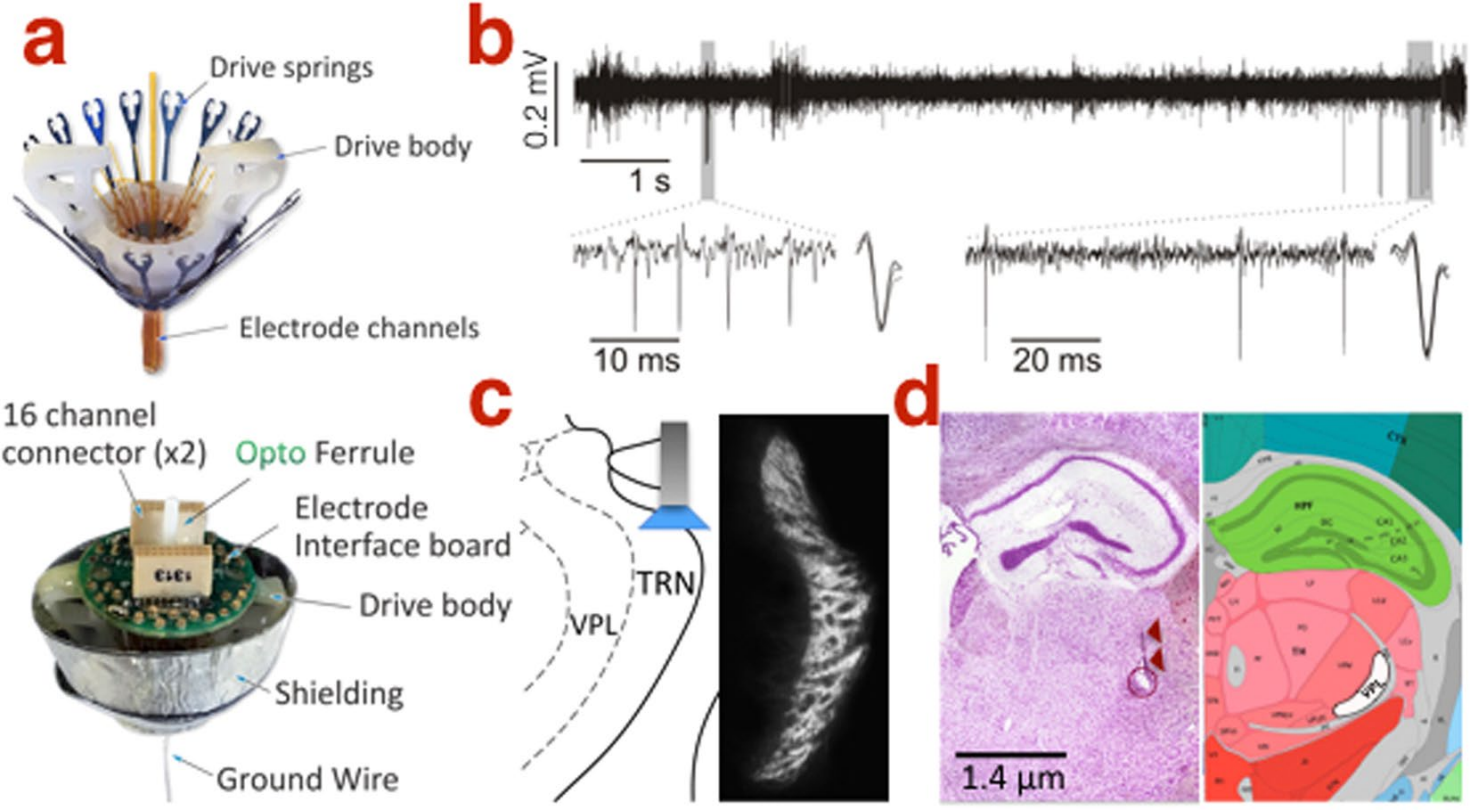
Extracellular *in vivo* recording. (**a**) Assembly of the FlexDrive stereotrode system mounted with a fiberoptic ferrule. (**b**) Isolation of two putative single-units from a 300–3000 Hz band-pass local field potential. (**c**) Channelrhodopsin-2 expression restricted to TRN in the VGAT mouse. (**d**) Representative coronal section showing electrolytic lesion (circle; arrows mark tetrode track) denoting a recording site in VPL (white shadow in right panel; Photo in 1d is adapted from the Allen Developing Mouse Brain Atlas; for reference see http://atlas.brain-map.org/atlas?atlas=1&plate=100960228#atlas=1&plate=100960228&resolution=18.60&x=5535.999348958333&y=4142.9498693678115&zoom=−4, and for copyright policy see https://www.alleninstitute.org/legal/citation-policy/).

To selectively induce thalamic bursts, we optically stimulated TRN neurons in awake, unrestrained transgenic mice (VGAT) co-expressing the vesicular GABA transporter with Channelrhodopsin-2 (ChR2). We used a custom-made multi-channel system^26^ to record extracellularly from putative single-units in ventral posterolateral (VPL) thalamus and LFP in SI hindlimb area^26–29^ (Fig. 1a,b). Histological analysis confirmed ChR2 expression limited to GABAergic neurons in TRN (Fig. 1c,d **;** additional criteria for tetrode localization shown in Supplemental Fig. 1).

**Figure 2:**
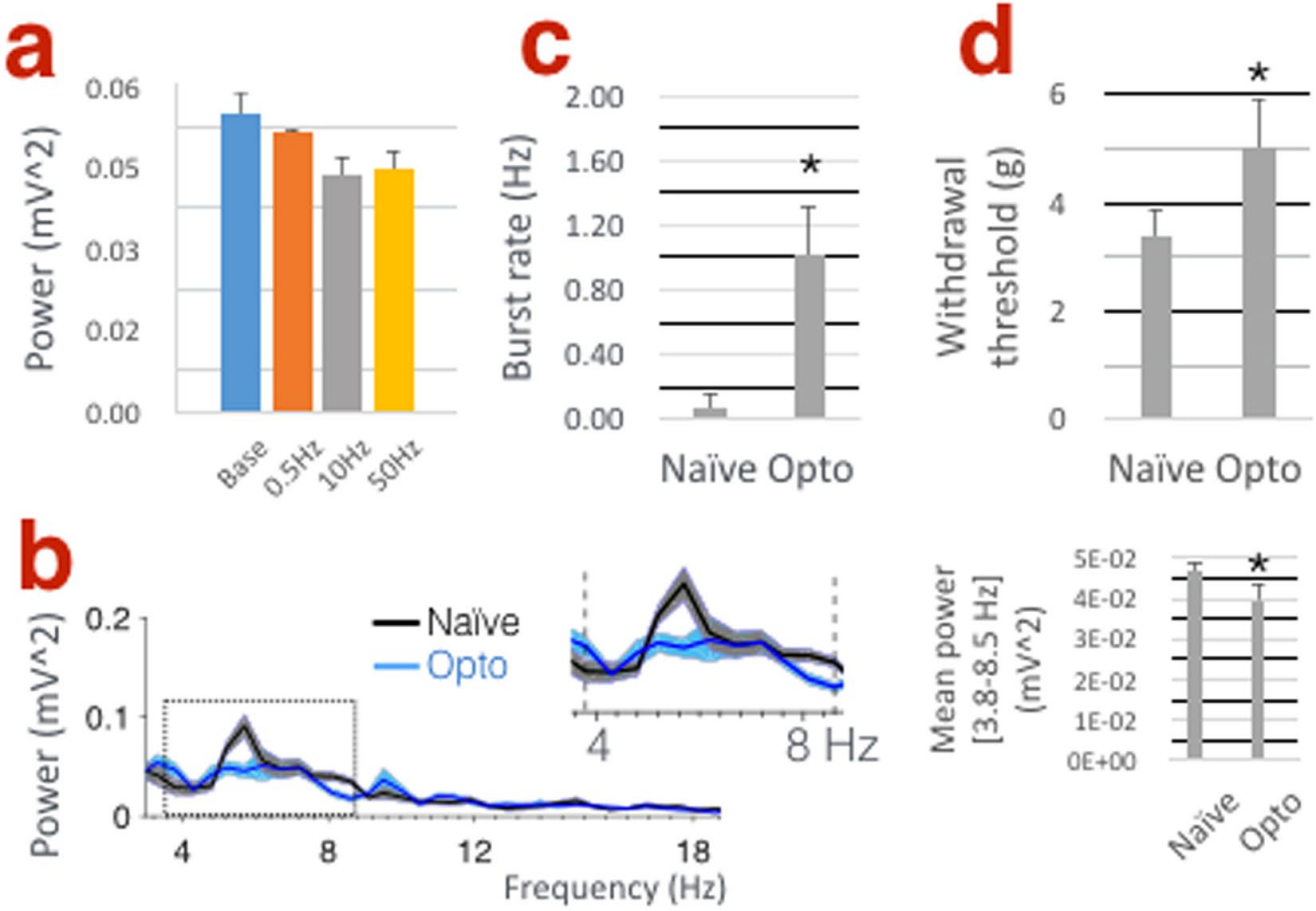
TRN stimulation decreases SI power in the theta band while increasing thalamic bursts and withdrawal threshold in naive VGAT mice. (**a**) Histogram showing the effects of TRN stimulation at 0.5, 10 and 50 Hz on mean theta (4–8 Hz) power under 1.5% isoflurane sedation (n = 2 mice). (**b**) SI power spectra. Inset in right panel shows significant decrease in power within the theta band (3.8–8.5 Hz) following 10 Hz TRN stimulation in awake mice (n = 5 mice). (**c**) TRN stimulation increases burst firing in VPL neurons (n = 17 units, 3–4 units per mouse; 5 mice) and increases the threshold of mechanical withdrawal to von Frey stimuli (**d**; n = 4 mice).

In a pilot experiment using naive VGAT mice, we confirmed that optical stimulation at 10 Hz (consistent with the physiological ‘baseline’ firing rate of TRN neurons^30^) effectively reduced SI theta power in sedated mice (Fig. 2a; TRN stimulation at 0.5, 10 and 50 Hz reduced SI theta power to 5.14 × 10^−2^ ± 0.71 × 10^−2^ mV^2^, 4.35 × 10^−2^ ± 0.33 × 10^−2^ mV^2^ and 4.45 × 10^−2^ ± 0.36 × 10^−2^ mV^2^ respectively compared to baseline 5.48 × 10^−2^ ± 0.38 × 10﻿^−^﻿^2﻿^ mV^2^). We then confirmed in awake, resting mice that TRN stimulation at 10 Hz reduces power within the theta range (3.8–8.5 Hz) (Fig. 2b; 4.70 × 10^−2^ ± 0.25 × 10^−2^ mV^2^ vs. 3.97 × 10^−2^ ± 0.40 × 10^−2^ mV^2^, P = 0.033). Moreover, TRN stimulation increased the burst firing rate of putative single-units in VPL (Fig. 2c, 0.07 ± 0.09 vs. 1.01 ± 0.31 Hz, P = 0.002) in accordance with previous studies by Moore and colleagues^27–29^ (see Supplemental Fig. 2 for the effects of TRN stimulation on tonic firing and the temporal distribution of VPL spikes; Supplemental Fig. 3 shows that the same stimulation paradigm in a wild-type mouse had no effect on theta power or thalamic firing rate). This stimulation also enhanced the threshold of paw withdrawal to von Frey stimuli (Fig. 2d; 3.38 ± 0.52 g vs. 5.02 ± 0.88 g, P = 0.03).

**Figure 3:**
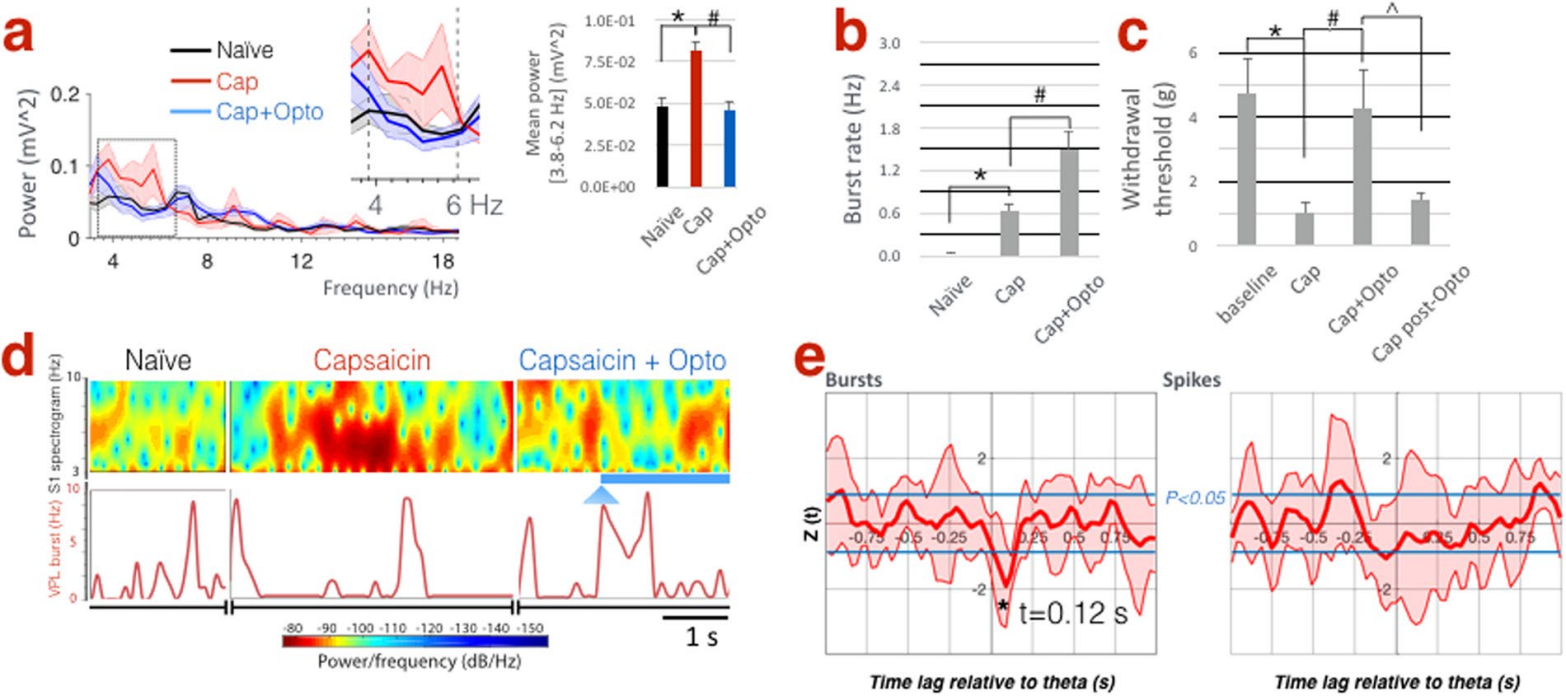
TRN stimulation during acute pain rescues SI theta power and reverses allodynia. (**a**) SI power spectra. Inset in right panel shows increased power between within the theta band (3.8–6.2 Hz) following capsaicin compared to naive, whereas TRN stimulation reverses these changes (n = 5 mice). (**b**) Capsaicin increases burst firing in VPL neurons, which is further enhanced following TRN stimulation (n = 17 units, 5 mice). (**c**) Withdrawal thresholds following capsaicin indicating tactile allodynia, which is reversed upon TRN stimulation but re-emerges 5 min afterwards (n = 7 mice, including 5 mice whose electrophysiological data are shown here). (**d**) Spectrogram illustrating the temporal dynamics of SI theta in relation to bursts in VPL under naive, capsaicin, and capsaicin + optogenetic conditions (arrowhead marks light onset; blue line marks duration of optical stimulation). Note theta and burst epochs do not temporally coincide. (**e**) Dynamic, time-lagged cross-correlation between SI theta power versus tonic and burst firing showing significantly negative correlation between theta-bursts when bursts precede theta by 120 ms (n = 17 units; 5 mice).

Next, we proceeded to testing our hypothesis in a pain model. As expected from our previous studies in rats^2–4^, SI power increased significantly in the theta band within 15–20 minutes after intradermal injection of capsaicin in the hindpaw (Fig. 3a; 3.8–6.2 Hz mean power 4.82 × 10^−2^ ± 0.57 × 10^−2^ mV^2^ vs. 8.15 × 10^−2^ ± 0.13 × 10^−2^ mV^2^, *P = 0.048). In these mice, TRN stimulation effectively reversed the pain-related increase in SI power to normal levels (Fig. 3a; 8.15 × 10^−2^ ± 0.13 × 10^−2^ mV^2^ vs. 4.55 × 10^−2^ ± 0.75 × 10^−2^ mV^2^, ^#^P = 0.002). The rate of spontaneous burst firing in VPL neurons increased after capsaicin injection and was further enhanced during TRN stimulation in the same neurons (Fig. 3b; 0.02 ± 0.02, 0.64 ± 0.11 and 1.50 ± 0.25, *P = 0.00002, ^#^P = 0.001; Supplemental Fig. 4 shows data related to tonic firing). As expected in this well-documented model of acute pain, paw withdrawal threshold decreased within 15–20 minutes after capsaicin injection (Fig. 3c; 4.47 ± 1.07 vs. 1.00 ± 0.37 g, *P = 0.011) suggesting mechanical allodynia, hallmark sign of neuropathic pain. Optical TRN stimulation, however, elevated withdrawal thresholds to near pre-capsaicin levels (Fig. 3c; 4.28 ± 1.22, ^#^P = 0.023) and reversal of these anti-nociceptive effects occurred within 5 min afterwards (Fig. 3c; 1.41 ± 0.22, ^P = 0.048). In previous studies^2, 3^, SI theta power was enhanced under acute and chronic pain conditions. Therefore, we further investigated the longitudinal effects of TRN stimulation on thalamic firing, theta power and nociceptive behavior following chronic constriction injury (CCI) of the sciatic nerve in the same animals. The results of these studies are comparable overall to those obtained in the capsaicin pain model (Supplemental Fig. 5), corroborating our previous observations^2, 3^.

We then investigated the temporal relation between thalamic firing and cortical theta. As shown in a representative example, epochs of high theta power and burst events do not coincide temporally (Fig. 3d; representative example showing time series of SI spectrogram with corresponding VPL burst rate). Therefore, we asked whether bursts preceded the decrease in theta power by a specific time lag. Dynamic, time-lagged cross-correlation between burst or tonic firing rate *versus* theta power revealed a significantly negative correlation between theta amplitude and burst rate, suggesting that bursts (but not tonic firing) are likely a trigger for the down-regulation of SI theta with a time lag of 120 ms (Fig. 3e).

Thalamus and cortex form mutually interdependent structures whose coordinated actions shape sensory experiences including pain. However, a conceptual framework or model that allows systematic testing of hypotheses regarding causality between thalamic unitary activity, cortical oscillations and pain has been elusive.

Cortical theta power is enhanced under pain conditions in rat models^2–4^ and in patients with chronic pain^1, 5, 6, 31^, but it is suppressed upon systemic administration of analgesics in rat pain models^2^ and therapeutic lesions in central lateral thalamus of pain patients^1, 5^. Overall, it has been postulated that high EEG power in the low-frequency range represents a neural correlate for pain^32^, and that thalamus plays a key role in the ‘thalamocortical dysrhythmia’ attributed to pain and other cognitive disorders^6, 33^.

Thalamic relay cells have long been known to fire in distinctive tonic or burst firing regimes that switch in dynamic and state-dependent manners^8, 34–36^. Our previous characterization of burst patterns in rat models of chronic pain^4, 12, 13, 17^ is consistent with clinical observations in pain patients^14, 16, 20^. We also reported that burst inhibition by intrathalamic injection of a T-type calcium channel antagonist attenuates cortical theta and nociceptive behaviors^4^. These prior studies demonstrated that burst inhibition is associated with a decrease in nociceptive behaviors using deep brain stimulation^13^, as well as *via* pharmacologic targeting of VPL that implicated molecular mechanisms involving voltage gated sodium^12^ and calcium channels^4^. Other studies have argued that thalamic bursting in fact *attenuates* somatic and visceral nociceptive behaviors in animal models^17–20^. Also, thalamic bursts have been observed in patients with neurological non-painful conditions^16^.

Suboptimal temporal resolutions imposed by pharmacological and structural approaches in the past have precluded a reliable inference to causality between these phenomena. In this study, the dynamic cross-correlation analysis provided a statistical construct for studying theta variability on short time scales which are reminiscent of EEG ‘microstates’ originally described as brief episodes of stability lasting in the order of 100 ms^37^. This fine spatiotemporal analysis of electrophysiological single-unit and LFP data in the thalamocortical network during wake state revealed that thalamic bursts in fact precede attenuation of SI theta power, leading to mechanical *hypo-*sensitivity. In contrast, thalamic tonic firing is not likely to be related to significant changes in theta power.

Thalamic bursts correlate with potent activation of cortical circuits^38^ and augmentation of visual detection^39^, suggesting a dynamic role in sensory processing. Although burst firing was regarded as absent in thalamic neurons and of no useful function during normal waking behavior^40^, evidence to the contrary is shown here and in other studies^9^ arguing for an important role in sensory transmission in the wake state. Though burst probability is indeed low during waking, frequent bursts could possibly be evoked by synchronous afferent volleys^10^, which is the case here following recruitment of TRPV1-expressing C-fibers upon capsaicin injection.

A limitation of this study is its focus on the VPL-SI network, which forms part of a more widely-distributed network underlying the conscious experience of pain. These results recommend future studies investigating spatiotemporal dynamics in thalamo-cortico-limbic structures involved in predictive coding of nociceptive ‘error’ signals^41^, as well as sensory behaviors of somatotopic or visceral origins. Moreover, our data showing increased tonic firing in response to TRN stimulation are distinct from results reported by Moore and colleagues regarding overall suppression of neurons in ventral posteromedian thalamus. Here, our choice of stimulation at 10 Hz corresponds to the physiological spontaneous firing rate of TRN neurons *in vivo* ^25, 30, 42^, noting that the effects of TRN stimulation in the current mouse model are known to be frequency-specific^27–29^. With regards to a mechanistic explanation for how spatiotemporal dynamics in the thalamocortical network can alter a nociceptive spinal reflex, we note a possible role for descending corticospinal projections such as from areas 3b, 1 and 2 of SI terminating in superficial laminae I-II of the dorsal horn, as well as projections from areas 3a and 4 terminating in deeper laminae III-V^43–45^. In rats, electrical stimulation in SI inhibits wide-dynamic range neurons in dorsal horn^46^, which is thought to be mediated by presynaptic inhibition of C-fiber afferents *via* the corticospinal tract^47, 48^. Electrical or pharmacological stimulation of sensorimotor cortex has also been shown to suppress noxious stimulus-evoked behavioral responses^49–51^. Finally, intricate connections between TRN, VPL and SI mandate precise locations of the recording and stimulating probes, whereby activation of multiple TRN neurons might potentially affect widespread cells in VPL and TRN trans- or multi-synaptically. Nonetheless, the consistency and reproducibility of our observations in many units recorded longitudinally over extended time periods, across multiple trials and from several animal subjects lend further validity to our results.

In summary, a work plan has been proposed by others to systematically investigate the role of thalamus in pain^52^. This plan included, among others, paired thalamocortical recordings, modulation of thalamocortical activity, and longitudinal assessment of pain-indicative behavior. We followed a similar approach here and used a selective neuromodulation technique in an established animal model, concluding that promotion of burst firing in thalamocortical neurons during naive and pain states is negatively correlated with cortical theta and mechanical allodynia. Notably, our current results corroborate a hypothetical framework we recently proposed regarding error signaling and predictive coding in the nociceptive circuitry^53^. We conclude that peripheral noxious stimuli evoke tonic firing and burst firing in thalamic neurons with upstream augmentation of cortical theta, whereby TRN plays an adaptive role in down-regulating theta and nociceptive behavior *via* thalamic bursts.

## Materials and Methods

### Animals

Transgenic mice co-expressing vesicular GABA transporter (VGAT) with Channelrhodopsin-2 (VGAT-ChR2-YFP) were purchased from Jackson Laboratory. In these mice, ChR2 expression in the thalamus is restricted to the thalamic reticular nucleus (TRN)^27^. Age-matched wild-type (C57 Bl\6 J) non-ChR2 expressing mice were also used to control for non-specific optical stimulation effects. Animals were housed individually, under a 12-hour light/dark cycle, in a temperature and humidity controlled environment with food and water available *ad libitum*. All the methods were carried out in accordance with the relevant guidelines and regulations and experiments were approved by the Rhode Island Hospital Institutional Animal Care and Use Committee. In the methods section Naive state refers to normal conditions prior to induction of the pain models.

### FlexDrive multi-channel recording

Thirty-two channel FlexDrive systems were assembled as previously described^26^; (www.open-ephys.org/flexdrive). Eight independently-manipulated tetrodes were built using 0.0005″ HFV-coated tungsten wire (California Fine Wire). Drives were positioned over the right side of the brain targeting VPL thalamus (Bregma −1.22 to −1.40, 1.75 to 2.00 lateral, 3 to 4 mm vertical) and SI cortex (Bregma −0.86 to −1.10, 1.5 to 1.8 lateral, <0.5 mm vertical). In each mouse, 3–4 tetrodes were positioned in VPL or SI and one tetrode in TRN, where an optical fiber was positioned over somatosensory TRN (Bregma −1.20 to −1.34, 2.30 to 2.40 lateral, 3.5 mm vertical) (see refs 27–29). FlexDrives were fixed to the skull using Metabond Adhesive (Parkell). After 3 days postoperatively, tetrodes were lowered incrementally (~500 µm over 5–7 days) until auditory confirmation of typical neuronal responses as expected in VPL and SI (for example increased multiunit firing) evoked by light brushing of the left hindpaw. Tetrode positions were also corroborated by stereotaxic coordinates, as perviously described^4, 11–13^. Additional criteria for identifying VPL units included peak-to-trough duration of the action potential^28, 29^, and the observations that most VPL neurons increase in firing rate in response to gentle brushing and noxious pinch of the receptive field (i.e. wide dynamic range type) while TRN neurons are predominantly inhibited^25, 52^. Chronic implants were stable over several weeks, allowing longitudinal analysis of neuronal activity with behavioral testing of mechanical sensitivity (watch video of the *in vivo* set-up https://www.youtube.com/watch?v=SSNpVuwIc6c).

### Electrophysiological recording in naturally behaving mice

Mice were briefly sedated (1% isoflurane <2 min) to allow connection of the FlexDrive to two-16 channels preamplifier (TDT RA16PA), headstages (TDT LP16CH) and a fibre optic patch cord (200 µm, Thor labs). Unrestrained mice later recovered from sedation in an 3 × 3″ plexiglass enclosure for at least 15 min prior to the start of electrophysiological recording using a TDT RZ2 BioAmp processor at 24.4 kHz sampling rate per channel. Two sequential notch filters (58–62 Hz) were applied to reduce electrical interference. The behavior of the animal was noted to determine alert rest periods, defined as alertness with no vigorous movements such as grooming or scratching. At the end of the final recording session, electrolytic lesions were performed for postmortem histological verification of recording sites, whereby brains were removed, immediately placed in cryogenic compound (OCT) and frozen at −80 C for further cryosectioning. Serial sections (25 µm) were treated with cresyl violet and hematoxylin for viewing under light microscope.

### Tonic and burst spike sorting

Extracellular spike waveforms (action potentials) in VPL were detected and sorted from LFP waveforms, bandpass filtered at 300–3000 Hz, using primarily template matching and principle component algorithms in Spike2 (CED 1401, Cambridge Electronic Design, UK). Sorted spikes were then screened visually and inspected for false-positive or overlapping unitary assignments. Only one electrode per tetrode was used for spike sorting to minimize redundant assignments from the same unit. Hence, 3–4 units were isolated from VPL per mouse, whereas cortical oscillations reflected the mean of 3–4 LFP measurements in SI. Moreover, isolation of putative unitary spikes also met the criterion of inter-spike interval (ISI) >2 ms (refractory period). Burst analysis was performed on sorted spikes as previously described by our laboratory^4, 11–13^ and others related to thalamic bursting evoked specifically by TRN stimulation^28, 29^, whereby burst events were identified according to the following parameters: Maximum interval signifying burst onset = 4 ms, offset = 8ms, longest increase in ISI within a burst = 2 ms, and minimum number of spikes within a burst = 2.

### Optical stimulation of TRN neurons

Laser light pulses were generated using a 100 mW 473 nm laser (MBL473 Opto Engine LLC) connected to the FlexDrive *via* fiber patch cord. Pulse control was achieved using an isolated pulse generator (A-M systems 2100) at a 10 Hz frequency, 0.5 ms pulse width, and total duration of 5 sec during electrophysiological recording. For behavioral testing of the mechanical withdrawal threshold, optical stimulation was applied for 2 sec during the application of von Frey filaments (see below).

### Acute and chronic pain models

Capsaicin (0.1%, 10 µl, intradermal, Sigma Aldrich) was injected into the plantar aspect of the left hindpaw under sedation (1.5% isoflurane <2 min) to prevent stress due to restraining the hindpaw. A TRPV1 agonist, capsaicin has been shown to cause increased neuronal firing of nociceptors, mainly polymodal C-fibers^54^, and is commonly used as a model of acute nociceptive pain in our laboratory^2, 3^. Chronic constriction injury (CCI), a well-documented model of chronic neuropathic pain^55^, was induced in the same mice that underwent capsaicin treatment 3 days post-injection after verifying that mechanical withdrawal returned to normal, as we previously reported^2–4, 13^. The sciatic nerve was exposed unilaterally after skin incision at the midthigh level and blunt dissection of the biceps femoris under deep anesthesia (isoflurane, 3.5%). Four chromic gut(5-0) ligatures were tied loosely around the nerve 1mm apart, and the overlying muscles and skin were closed in layers with 5-0 Ethilon sutures.

### Data Analysis

#### Power

Fast Fourier transform function (“fft”) was used to convert LFP waveform from the time domain to the frequency domain, yielding power spectral density (PSD) histograms using 5 sec time intervals during awake resting state (no difference was found compared to the multi-taper method). Values were generated at 57 frequencies (0.47 Hz bins) between 3–30 Hz. For the pain state, data were collected within 15–20 min after capsaicin injection.

#### Mechanical withdrawal threshold

Mechanical sensitivity of the hindpaw was assessed by measuring the threshold of withdrawal in response to the application of calibrated von Frey filaments of different bending forces to the plantar aspect of the hindpaw according to the ‘up-down’ method^56^, whereby filaments of different bending forces were pressed against the paw until buckling for a maximum of 3 sec or a withdrawal reflex. This well-documented test represents naturally-occurring stimulation to the hindpaw in the noxious and non-noxious range evoking a biologically-relevant state in mammals^57^.

#### Conditioned place preference

In the dual chamber conditioned place preference (CPP) test, as originally described^58^, FlexDrive-implanted mice were conditioned with unrestricted access to both chambers for three days, with baseline preference determined on the third day. On the fourth day, mice underwent ‘pairing’ by being individually restricted to one chamber and receiving optical stimulation (10 Hz, 0.5 ms pulse width) for 30 sec, then 4 hours later they were restricted to the opposite chamber for 30 min after receiving optogenetic stimulation. On the fifth day, mice were allowed free access to both chambers. Chamber preference was video recorded and analyzed off-line by an observer blinded to the animal’s treatment.

#### Cross correlation

The distribution of the number of bursts and spikes in VPL per bin, and the magnitude of SI theta power per bin were analyzed for 919 bins for each mouse (n = 5, bin size 30 ms). Regarding SI theta power, the mean observed power of 3 consecutive bins was used as the representative power of a bin (*e.g*. the average of the observed power of the bin_i−1_, the bin_i_, and the bin_i+1_ was used as the representative power of the bin_i_) to satisfy the conditions of accurate power estimation (100 ms bin size) and fine temporal resolution (30 ms bin size). Analysis revealed both the number of bursts and spikes *per* bin had Poisson distribution and more than one burst or two spikes per bin were considered significant events, and that SI theta power per bin had lognormal distribution. Therefore, the relationship between fluctuation of SI theta power and spikes or bursts was analyzed using cross-correlation analysis as described previously^59–61^. Briefly,

1 $${\rm{Q}}({\rm{t}})=\,1/(T-t)\sum _{i=1}^{T-t}X(i)Y(i+t)$$

was calculated as previously described^60^. Where, in the case of burst, *X(i)* was 1 (if there were any bursts in the bin_i_) or 0 (if there was no burst in the bin_i_), and in the case of spikes, *X(i)* was 1 (if there were more than two spikes and no burst in the bin_i_) or 0 (otherwise). *Y(i* *+* *t)* represented the fluctuation of theta power with t bins lags from the bin_i_, and was calculated as follows

2 $${\rm{Y}}({\rm{i}})=df(i)/di=\,\{f(i+1)-f(i)\}/{\rm{\Delta }}i$$

where *f*(*i*) represents “–log transformed S1 theta power at bin_i_”, and Δ*i* is the size of bin_i_. If no relationship is found between bursts or spikes in VPL and fluctuation in SI power, *Q*(*t*) would have normal distribution. Thus, Z value was calculated for each *Q*(*t*) as follows:

3 $${\rm{Z}}({\rm{t}})=\{Q(t)-E[Q(t)]\}/\sqrt{V[Q(t)]}$$

where

4 $${\rm{E}}[{\rm{Q}}({\rm{t}})]={\rm{E}}[{\rm{X}}]{\rm{E}}[{\rm{Y}}]$$

and

5 $${\rm{V}}[{\rm{Q}}({\rm{t}})]=1/(T-t)(E[{X}^{2}]E[{Y}^{2}]-{\rm{E}}{[X]}^{2}E{[Y]}^{2})$$

Z(t) was calculated for each mouse, and then, the average of Z(t) and 95% confidence interval of Z(t) were calculated (see ref. 61).

### Statistics

Analysis of variance (ANOVA) and parametric tests were used. Two-way ANOVA analysis followed by Bonferroni’s correction, Student’s *t*-test, or z-score method was used to compute statistical significance as described in text. Bartlett’s test was performed to compute normal distribution and equal variance. A P value < 0.05 was considered significant (denoted with * in figures). For behavioral and power data, comparisons were made between animal groups and for spike and burst activity data comparisons were made between neuronal groups. All values are reported as ± standard error of the mean (SEM).

## Electronic supplementary material

Supplementary Information
