# Supplementary material for: Thalamic Bursts Down-regulate Cortical Theta and Nociceptive Behavior
Source: Sci Rep. 2017 May 30;7:2482. doi: 10.1038/s41598-017-02753-6 (PMC5449396; doi:10.1038/s41598-017-02753-6)
Supplement: Supplementary file 1 — Supplementary Information [file 41598_2017_2753_MOESM1_ESM.doc]

# Supplemental information

# Title: Thalamic Bursts Down-regulate Cortical Theta and Nociceptive Behavior

Brian W. LeBlanc ^1,2^, Brent Cross ^1,2^,, Kelsey A. Smith ^1,2^, Catherine Roach ^1,2^, Jimmy Xia ^1,2^, Yu-Chieh Chao ^4^

Joshua Levitt ^3^, Suguru Koyama ^1,2,5^, Christopher Moore ^2^, Carl Saab* ^1,2^

1 Department of Neurosurgery, Rhode Island Hospital, Providence, RI, USA

2 Department of Neuroscience, Brown University, Providence, RI, USA

3 Center for Biomedical Engineering, Brown University, Providence, RI, USA

4 Department of Anesthesiology, Beijing Chaoyang Hospital, Capital Medical University, Beijing, China

5 Laboratory for Pharmacology, Asahi KASEI Pharma Corporation, Shizuoka, Japan

**Corresponding author address:*

*Rhode Island Hospital, 593 Eddy St, Providence, RI 02903, USA. E-mail address: carl_saab@brown.edu (C.Y. Saab).*

***
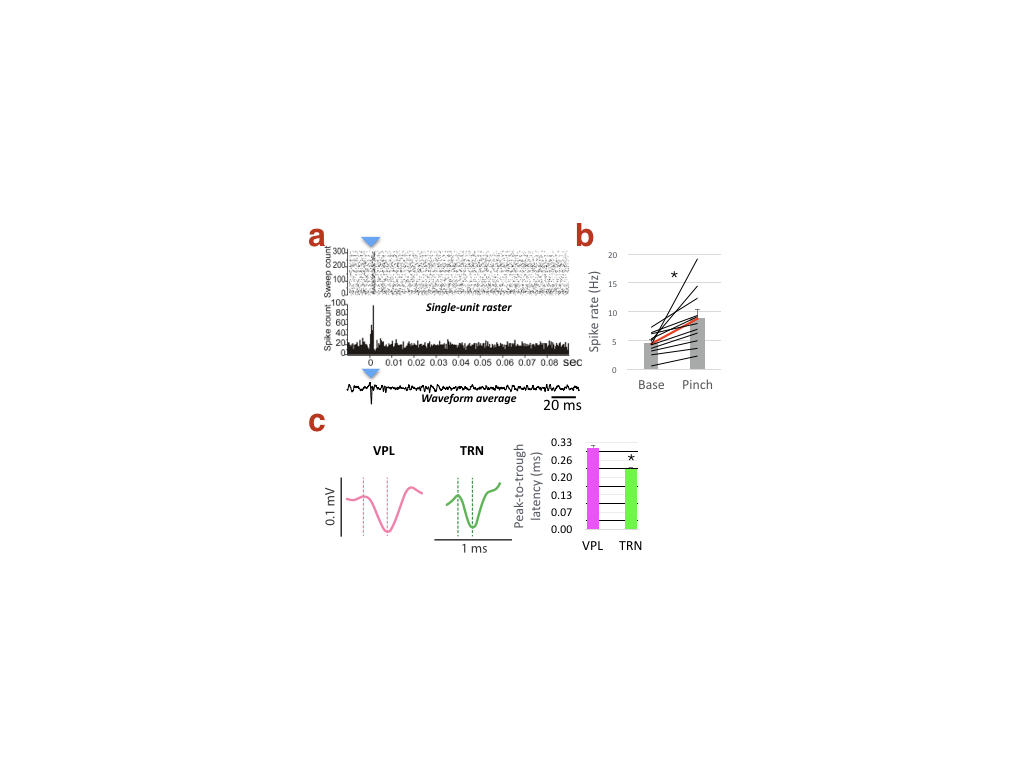
***

***Supplemental Figure 1:*** Extracellular *in vivo* recording in TRN and VPL. a) Raster plot showing time-locked firing in a putative TRN single-unit and an evoked response in the corresponding field potential to optical stimulation in the lateral somatosensory division of TRN at 10 Hz (arrowhead). b) Increase in putative single-unit firing in the somatosensory hindpaw area of VPL evoked by pinch stimuli applied to the receptive field on the contralateral hindpaw under light isoflurane sedation (baseline vs. pinch 4.6±0.5 and 8.9±1.5 Hz, P=0.006, n=11 neurons). c) Example showing off-line analysis of peak-to-trough spike latencies revealing distinct ‘wide' and ‘narrow’ profiles corresponding to VPL and TRN (VPL vs. TRN 0.31±0.01 and 0.23±0.01 ms, respectively, P=0.0007, n=11 units).

***
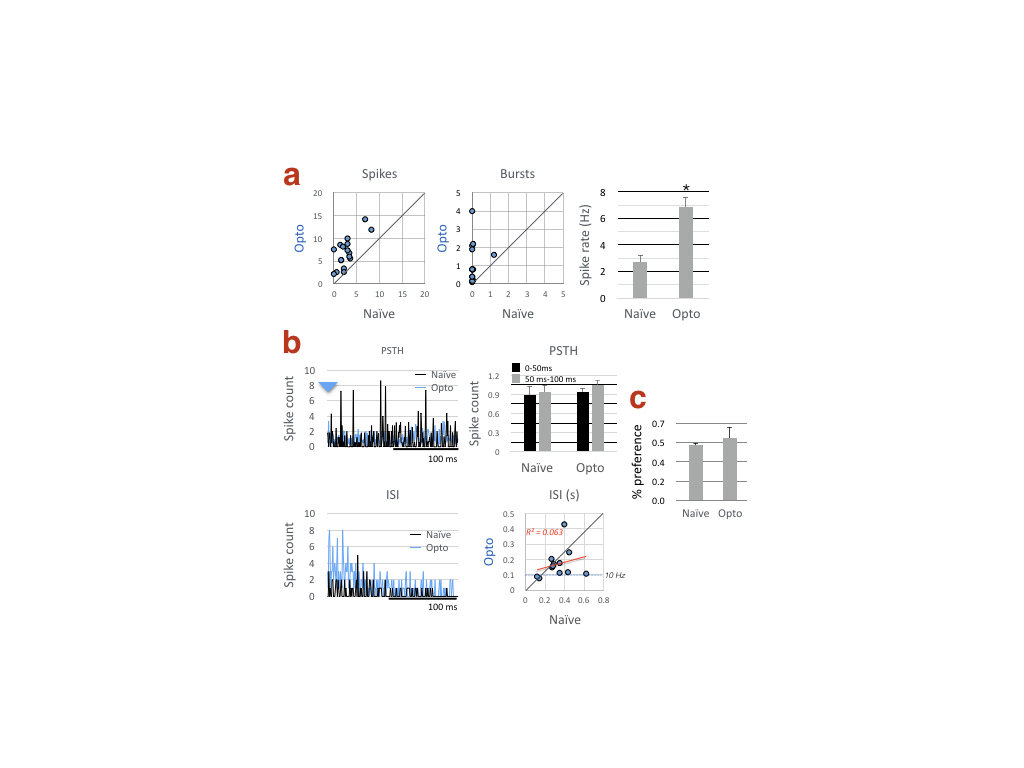
***

***Supplemental Figure 2:*** TRN stimulation enhances tonic and burst firing in thalamic neurons of naive VGAT mice and modulates the inter-spike interval (ISI) but does not affect the peri-stimulus time histogram (PSTH) or place preference. a) TRN stimulation increases tonic and burst firing (corresponding burst histogram shown separately in Fig 2) in VPL neurons (2.71±0.52 vs. 6.84±0.81 Hz, P=6x10^-6^, n=17 units, 5 mice). b) PSTH showing no change in spike count triggered by TRN stimulation (arrowhead), however, a leftward shift in ISI was noted. c) TRN stimulation has no effect on conditioned place preference (50±2 vs. 56±10 %, P=0.36, n=3 mice).

***
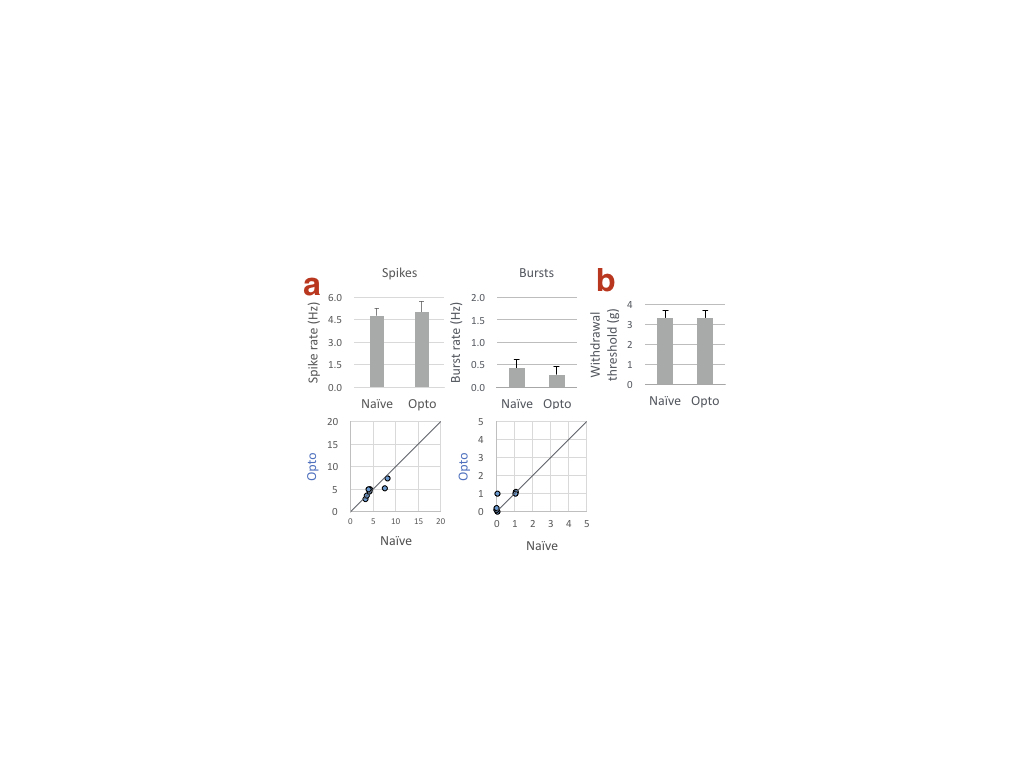
***

***Supplemental Figure 3:*** Optical stimulation in TRN has no effect on thalamic firing or withdrawal threshold in a naive non-ChR2 expressing wild-type mouse. a) Optical stimulation does not change tonic (4.78±0.55 vs. 5.03±0.76 Hz, n=7 units) and burst firing (0.430.20± vs. 0.29±0.18 Hz, n=7 units) in VPL neurons, or b) withdrawal threshold (3.34±0.41 vs. 3.34±0.41 s).

***
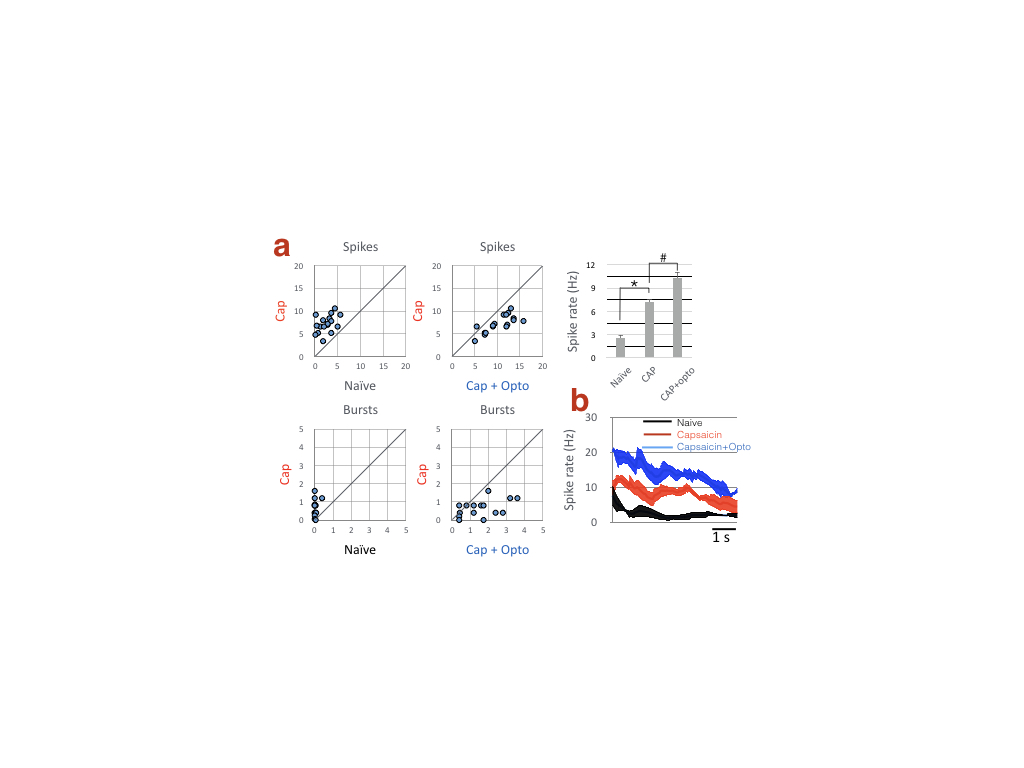
***

***Supplemental Figure 4*:** Effects of Capsaicin and TRN stimulation on tonic and burst firing in VGAT mice. a) Capsaicin increases tonic and burst firing (corresponding burst histogram shown separately in Fig 3) in VPL units, which are further enhanced following TRN stimulation (2.52±0.41, 7.19±0.46 and 10.32±0.76 Hz, *P=2x10^-8^, ^#^P=0.00001, n=17 units, 5 mice). b) Representative example showing increase in spontaneous firing rate following capsaicin in VPL neurons, and further enhancement following optical TRN drive (n=4 units, 1 mouse).

***
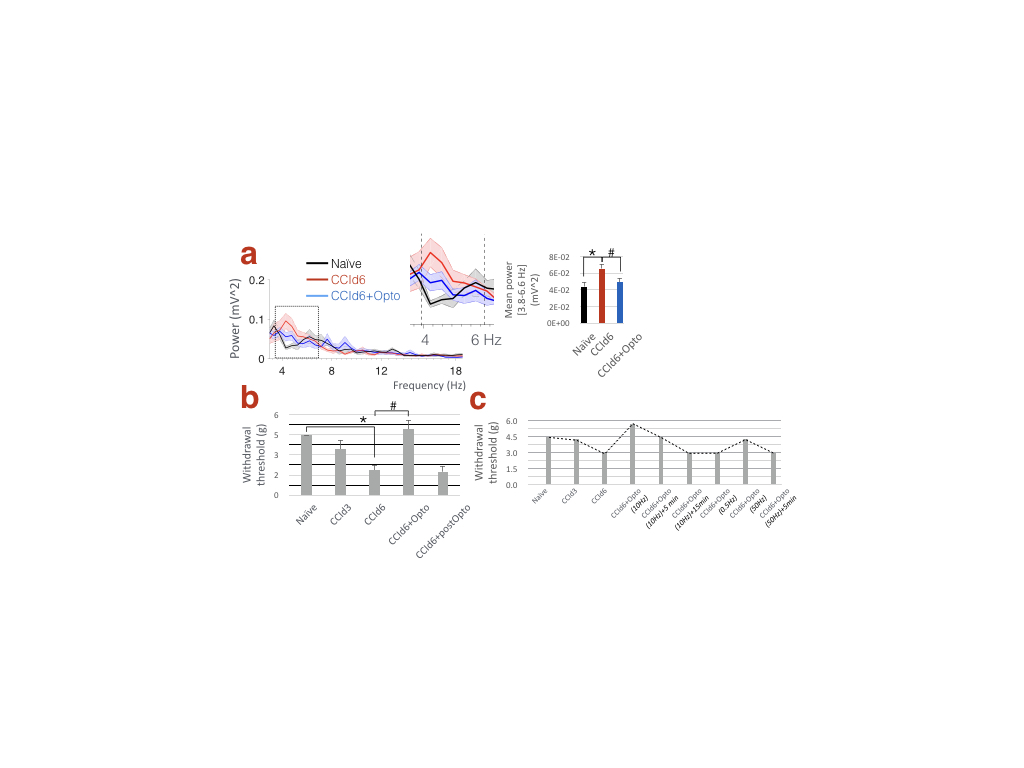
***

***Supplemental Figure 5:*** TRN stimulation during chronic pain rescues SI theta power and reverses allodynia. a) Mice with chronic constriction injury (CCI), a model of neuropathic pain, manifest increased SI power between 3.8-6.6 Hz at 6 days following CCI compared to naive (4.40x10^-2^±0.54x10^-2^ mV^2^ vs. 6.56x10^-2^±0.26x10^-2^ mV^2^, *P=0.028, n=5 mice; note a similar trend in the acute pain model following capsaicin injection), whereas TRN stimulation reverses these changes (6.56x10^-2^±0.26x10^-2^ mV^2^ vs. 4.89x10^-2^±0.21x10^-2^, ^#^P=0.007; n=5 mice). b) Withdrawal thresholds indicating tactile allodynia 6 days after CCI, which is reversed upon TRN stimulation but re-emerges 15 min afterwards (naïve 4.47±0.04 s, CCId3 3.59±0.74 s, CCId6 2.13±0.39 s, CCId6+Opto 5.29±0.36 s, CCId7+Opto(+15min) 1.92±0.53 s, *P=0.002, ^#^P=0.01, n=3 mice). c) Representative example from 1 mouse showing longitudinal analysis of paw withdrawal latency. Note the development of mechanical allodynia 6 days after CCI and rescue thereof upon TRN stimulation at 10 and 50 Hz, but not 0.5 Hz, suggesting frequency-specific anti-nociceptive effects.
